# Finite-temperature scaling of trace distance discord near criticality in spin diamond structure

**DOI:** 10.1038/srep42360

**Published:** 2017-02-15

**Authors:** W. W. Cheng, X. Y. Wang, Y. B. Sheng, L. Y. Gong, S. M. Zhao, J. M. Liu

**Affiliations:** 1 Institute of Signal Processing & Transmission, Nanjing University of Posts and Telecommunication, Nanjing, 210003, China; 2 National Laboratory of Solid State Microstructures & Innovation Center of Advanced Microstructures, Nanjing University, Nanjing, 210093, China

## Abstract

In this work we explore the quantum correlation quantified by trace distance discord as a measure to analyze the quantum critical behaviors in the Ising-XXZ diamond structure at finite temperatures. It is found that the first-order derivative of the trace distance discord exhibits a maximum around the critical point at finite temperatures. By analyzing the finite-temperature scaling behavior, we show that such a quantum correlation can detect exactly the quantum phase transitions from the entan-gled state in ferrimagnetic phase to an unentangled state in ferrimagnetic phase or to an unentangled state in ferromagnetic phase. The results also indicate that the above two kinds of transitions can be distinguished by the different finite-temperature scaling behaviors. Moreover, we find that the trace distance discord, in contrast to other typical quantum correlations (e.g., concurrence, quantum discord and Hellinger distance), may be more reliable to exactly spotlight the critical points of this model at finite temperatures under certain situations.

For a quantum many-body system, the ground state properties may undergo qualitative and dramatic changes owing to quantum fluctuations at zero temperature. This phenomenon, known as a quantum phase transition (QPT), is attributed to the interplay between the different orders associated with competing interactions in the Hamiltonian^1^. However, the QPT can also emerge and be observed at sufficiently low temperature if thermal fluctuations are not sufficient to drive the system away from its ground state to excited states. In the other words, the quantum fluctuations still dominate at these temperatures. Recently, the finite-temperature properties of QPT have been attracting attention due to the fact that all experiments are confined to finite temperature^2^,^3^,^4^. Thus, an understanding of only the zero-temperature properties of a quantum system is not sufficient from the perspective of experimental results.

On the other hand, quantum correlations among the subsystems of a many-body system are closely related to the emergence of the QPT. In recent years, such relationships have been studied from many different perspectives in varies quantum systems^5^,^6^,^7^,^8^,^9^,^10^,^11^,^12^,^13^,^14^,^15^,^16^,^17^,^18^,^19^,^20^,^21^,^22^. For instance, entanglement has been widely employed to identify QPT with great success^7^,^8^,^9^,^10^,^11^,^12^,^13^,^14^,^15^,^16^,^17^,^18^,^19^,^20^. However, entanglement may fail to measure the quantum correlations for a state in some specific cases (e.g., the case with remote spin pairs in a spin chain system^8^). That’s to say, there exist other quantum correlations which can not be grasped by entanglement. In addition, entanglement might signal a pseudo transition point^18^. These facts stimulate many works to classify and quantitate the quantum correlations from other perspectives in order to avoid this disadvantage^23^,^24^,^25^,^26^,^27^,^28^,^29^. In particular, Ollivier and Zurek introduced the so-called quantum discord (QD), which is based on the fact that two equivalent ways to define the mutual information in classical world turn out to be inequivalent in the quantum ones, in order to quantify all nonclassical correlations among quantum systems^23^. And QD has been attracted much attention in many branches of physics. One important aspect is the relationship of QD with QPT^30^,^31^,^32^,^33^,^34^,^35^,^36^,^37^,^38^. Moreover, Werlang *et al*. found that QD can characterize exactly the critical points of the XXZ Heisenberg chain even at finite temperatures, while entanglement seems not^39^. Unfortunately, an analytical solution to QD is known only for typical two-qubit states. Subsequently, many distance-based quantum correlation measures have been proposed, such as geometric quantum discord defined via the Hilbert-Schmidt distance^24^ (which may be changed by trivial local actions on the unmeasured party^25^) and its modified version via the Hellinger distance^26^,^27^. Another important version is the trace distance discord^28^, which is defined through the Schatten one-norm. This quantum correlation exhibits some attractive features, and thus can be a physically meaningful measure. For instance, the trace distance obeys a contractive property, owing to the definition in terms of the Schatten one-norm, and this property is invariant under the unitary transformation. Recently, Ciccarello *et al*. showed that the trace distance discord can be analytically obtained for an arbitrary *X* state^29^. Taking account of the great success and flaw of previous quantum correlations in detecting QPT, it is meaningful to investigate the behavior of the trance distance discord for a typical quantum critical system, so that the capability and advantages of this measure to detect the QPT can be tested.

Regrading the QPT itself, the low-dimensional frustrated quantum spin models with competing interactions have attracted considerable attention due to their attractive quantum critical behaviours. For instance, the spin-1/2 quantum Heisenberg model with diamond chain structure is actively engaged in the investigations of geometric frustration^40^,^41^,^42^,^43^,^44^,^45^. Interestingly, this quantum spin model can be employed to explain the properties of some real materials such as azurite which has the 1/3 magnetization plateau and exhibits the double peaks in both magnetic susceptibility and specific heat^45^. On the other hand, the Ising-XXZ diamond model can also provide an excellent ground for rigorous study of pairwise quantum correlations at finite temperature in an infinite chain structure. The goal of this work is to check whether the trance distance discord as a measure can be used to describe the quantum critical behaviors in the spin diamond structure at finite temperature. The main properties of the quantum criticality(e.g., the finite-temperature scaling behavior, universality and critical exponents) will be visited both numerically and analytically.

## Results

### Ising-XXZ model and ground state phase diagram

**Figure 1:**
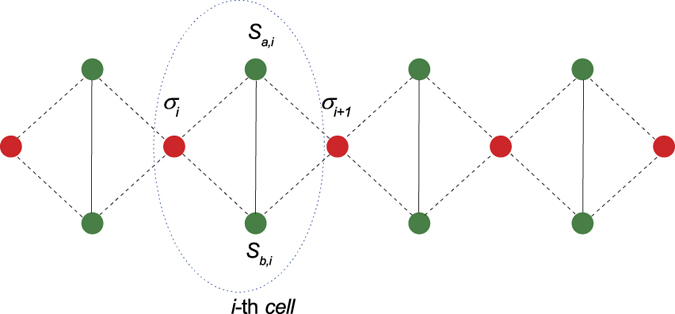
The schematic picture of Ising-XXZ diamond chain. The solid (dash) lines denote the Heienberg XXZ (Ising) interactions between two spins. The red (oliver) circles denote the Ising (Heisenberg) spins.

The Ising-XXZ model with interstitial anisotropic Heisenberg spins and mixed nodal Ising spins on a diamond-structure chain in the presence of an external magnetic field is illustrated in Fig. 1. The Hamiltonian operator can be expressed as follows^40^,^41^,^42^,^43^,^44^:


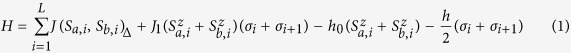


here, 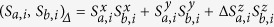 denotes the Heisenberg dimer interaction, Δ is the anisotropy parameter, *S*_*α,i*_ stands for the quantum Heisenberg spin operators of the *i*-th cell along the chain, *α* = *a,b* numbers the two sites of the Heisenberg dimer, 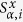,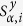 and 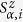 are three components of the Pauli operator, respectively. *σ*_*i*_ = ±1 denotes the classical Ising spin, while *h* and *h*_0_ are the external magnetic fields acting on Ising spins and Heisenberg spins, respectively. The parameters *J*_1_ and *J* correspond to the coupling constants of Ising interaction and XXZ interaction, respectively. For convenience, we set *J*_1_ = 1, *J* = 1 and *h*_0_ = *h* throughout this work. *L* is the number of cells in the chain and will be treated as infinite. The Heisenberg spin coupling can be expressed using matrix notation as following,


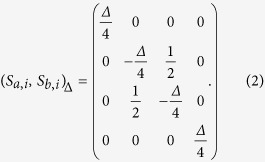


By fixing the values for *σ*_*i*_ and *σ*_*i*+1_, we can obtain the eigenstates in terms of basis {|↑↑〉, |↑↓〉, |↓↑〉, |↓↓〉} given by,


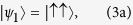



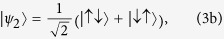



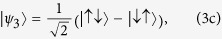



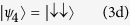


And the corresponding eigenvalues are 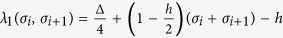, 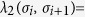

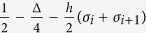
, 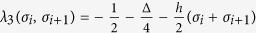, and 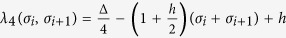, respectively. In earlier work, three magnetic phases were observed (frustrated phase, ferrimagnetic phase and ferromagnetic phase) for the system^40^. In the present study, we take the tactic proposed by Rojas *et al*., to re-arrange these phases into two main regions (entangled and unentangled)^41^, which are closely related to the above three different magnetic phase, i.e., entangled state in the frustrated phase, 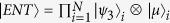, unentangled state in the ferrimagnetic phase 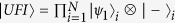 and unentangled state in the ferromagnetic phase 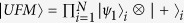. Here, |*μ*〉_*i*_ stands for an arbitrary value (*μ* = ±1/2) of the nodal spin in the *i*th block. According to the Bell states given in Eqs (3b) and (3c), at zero temperature the entangled state in the frustrated state is fully spanned at zero magnetic field, while the ferrimagnetic and ferromagnetic state are spanned by the unentangled states, respectively. The eigenvalues for the UFM is given by *E*_*UFM*_ = 1 + Δ/4 − 3*h*/2 and the eigenvalue for UFI state is *E*_*UFI*_ = −1 + Δ/4 − *h*/2. The entangled state (quantum ferrimagnetic state), denoted by 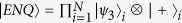, has its eigenvalue *E*_*ENQ*_ = −1/2 − Δ/4 − *h*/2. Thus, the boundary between these critical phases at zero temperature can be exactly figured out according to these eigenvalues.

### Trace distance and QPT at finite temperature

**Figure 2:**
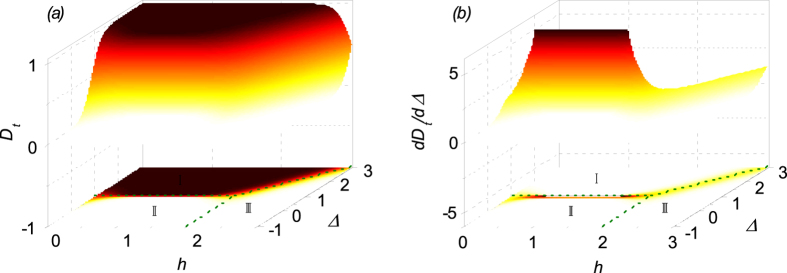
(**a**) The trance distance discord 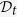 and (**b**) its first-derivative 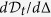 with respect to Δ and *h* under temperature *T*=0.1. In the ENQ phase, the value of 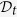 almost equals to one and it approaches asymptotically to zero in the UFI and UFM phases at low temperature. Around the critical lines, Δ = 1(*h* < 2.0) and Δ − 2*h* + 3 = 0(*h* > 2), and then the first-derivative 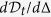 exhibit a maximal, marking a QPT at finite temperature.

It is noted that the thermal entanglement for such a model on different critical phases was once discussed in the previous works^41^,^42^,^43^,^44^. The results showed that the entanglement may disappear as temperature exceeds a threshold *T*_*c*_, making the entanglement fail to characterize the critical points above the critical temperature. Here we focus on the relationship between the trace distance discord and the quantum critical phenomenon at finite temperature. Without loss of generality, we plot the 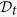 as a function of Δ and *h* for a fixed finite temperature *T* = 0.1 as an example in Fig. 2(a). Obviously, one can see that the UFI and ENQ regions (or phases) are separated by line Δ = 1 with *h* < 2, and the UFM and ENQ regions (or phases) are separated by line Δ − 2*h* + 3 = 0 with *h* > 2. The trance distance discord almost equals to one in the ENQ region and approaches to zero in the other two regions. Around the boundary between the unentangled state and entangled one, the trace distance discord falls quickly and approaches to zero asymptotically. Here it should be mentioned that the quantum state for Heisenberg dime is the unentangled state |*ψ*_1_〉 and thus all quantum correlations(concurrence, quantum discord, trance distance discord, etc.) equal to zero when the system is in the UFI or UFM phase. In the other words, these is no change for these correlations when the system goes across the critical line between the two phases. In Fig. 2(b), we plot the first-derivative of 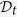 with respect to Δ for the fixed finite temperature *T* = 0.1. Again and obviously, the maximal appears around the critical lines. It is known that the first-derivative with respect to the driving parameter shows an extremal behavior near these critical lines and will be divergent in the thermodynamic limit *T* → 0.

To this stage, one is convinced that these properties of the trance distance discord indeed can reveal the critical regions at finite temperature for this model. In the following, we shall proceed to perform quantitative analysis on the 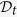 around the critical lines at finite temperature.

### Quantum criticality from ENQ to UFI

**Figure 3:**
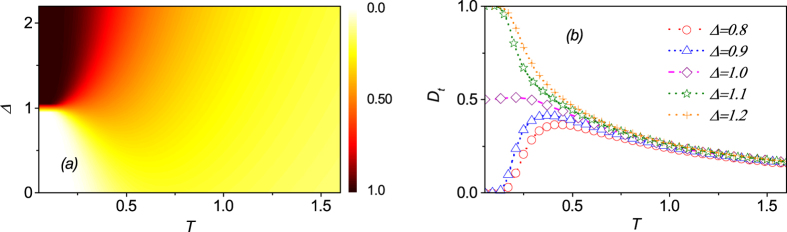
(**a**) The trance distance discord 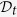 as a function of *T* and Δ. (**b**) The trance distance discord 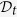 as a function of *T* at different values of parameter Δ.

Given the discussion above, it is clear that there exists a transition from ENQ phase to UFI phase around line Δ = 1 with *h* ≤ 2. In Fig. 3(a) is plotted the the density of 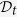 as a function of *T* and Δ for a fixed *h* = 1. Clearly 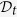 does not equal to zero over the whole parameter region and it is finite even at high temperature where the entanglement may disappear^41^,^44^. As addressed above, the reason is that 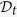 can measure the total quantum correlations of a state *ρ* while the entanglement can only reflect part of them. This property of 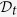 makes it possible to detect the quantum critical point at finite temperature. We also note that 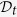 may increase with temperature in a given region, which is very similar to QD in the Heisenberg XYZ model^46^. Naturally, this character tends to disappear as temperature goes too high. In Fig. 3(b), 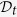 as a function of temperature *T* for several different values of Δ are plotted. Obviously, for Δ > 1, *D*_*t*_ decreases monotonically with increasing *T* and approaches to zero asymptotically in the high temperature region. However, for Δ < 1, 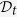 almost equals to zero in the low temperature region, and then increases up to a maximal before going down to zero asymptotically as *T* increases further. These results also indicate that there indeed exists a transition when the system goes across Δ = 1.

**Figure 4:**
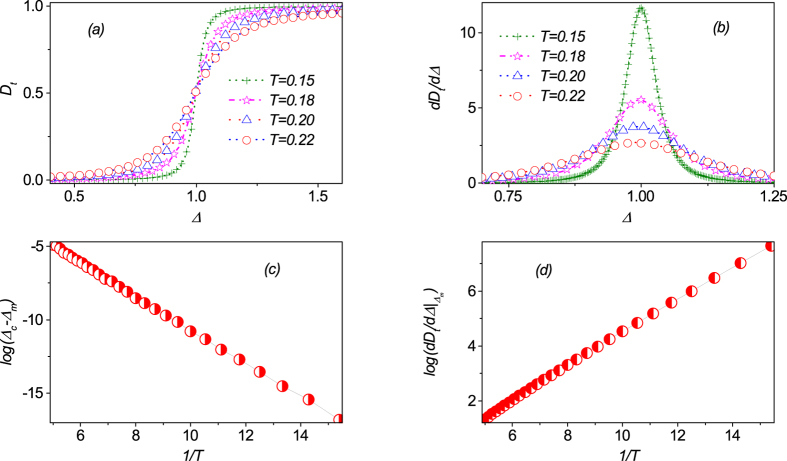
The calculated 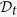 (**a**) and its first-derivative 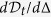 (**b**) as a function of parameter Δ around the critical point Δ = 1.0. (**c**) The peak position Δ_*m*_ can be regarded as a pseudo-critical point which shifts with increasing temperature *T* following relationship 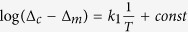 in approaching to the critical point Δ_*c*_. This behavior implies that Δ_*m*_ → Δ_*c*_ as *T* → 0. (**d**) The maximum value of 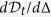 at the pseudocritical point Δ_*m*_ as a function of *T*.

To explore the effect of finite-temperature on 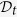, we present the calculated 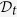 as a function of Δ at several temperatures *T* around the point Δ = 1.0 in Fig. 4(a). 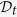 increases monotonically with increasing Δ and this trend becomes more significant as temperature *T* is lower. We plot the first-derivative of 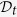 with respect to Δ in Fig. 4(b) to reflect such trend. The 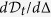 exhibits a clear singularity around the critical point Δ = 1.0 in the limit *T* → 0. Generally speaking, the appearance of nonanalytic behavior of a physical quantity is fundamentally a feature of QPT. It is accompanied with a scaling behavior due to the divergency of the correlation length. Fig. 4(b) shows the shift of the anomaly position, marked by the sharp peak, and the peak height with increasing temperature. In details, the peak position Δ_*m*_ can be regarded as a pseudo-critical point which shifts with temperature *T* following the scaling law:


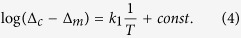


The numerical results are plotted in Fig. 4(c), implying Δ_*m*_ → Δ_*c*_ as *T* → 0. On the other aspect, the value of the derivative of 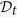 is logarithmically divergent at the pseudo-critical point Δ_*m*_ in the thermodynamic limit,


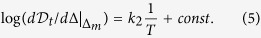


The numerical results are plotted in Fig. 4(d), suggesting that 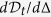 shows a singularity at the critical point as temperature approaches to zero.

**Figure 5:**
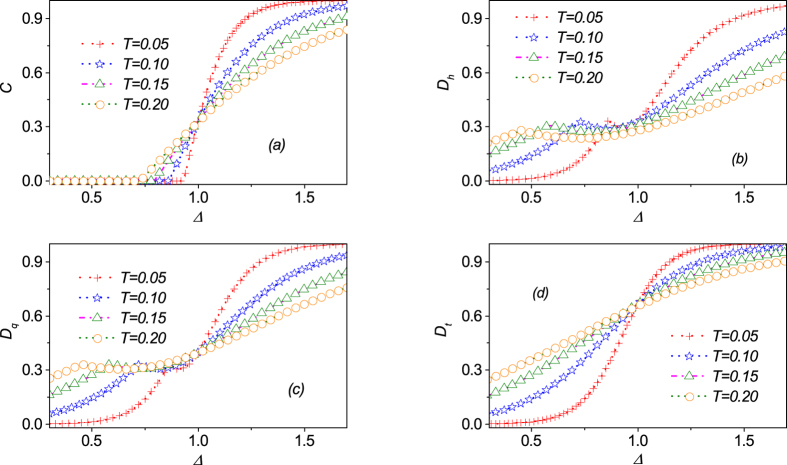
Several typical quantum correlations, (**a**) concurrence 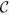, (**b**) Hellinger distance 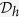, (**c**) quantum discord 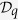, and (**d**) trance distance discord 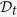 as a function of parameter Δ around the points(Δ,*h*) = (1,0) at various temperatures *T*, respectively. Obviously, they are all smooth functions of Δ. However, the first-derivatives for 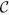, 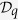 and 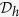 would exhibit an unexpected discontinuity point Δ_*f*_, which is unfortunately not a real critical point except normal discontinuity point Δ_*c*_ for the QPT.

As a comparison, it is rather interesting to check the behaviors of some other typical quantum correlations around the critical point. In Fig. 5(a), we plot concurrence 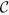 as a function of Δ at several different temperatures *T* with *h* = 0. Although the 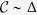 curves are continuous, there would exist two points where the corresponding first-derivative 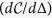 is continuous instead of divergent. The discontinuity at Δ_*c*_ = 1 does indicate the QPT of the present model. However, the unexpected discontinuity occurs at Δ_*f*_ where entanglement 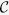 disappears, suggesting that this is a false critical point. This behavior for entanglement 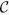 is very similar to the counterpart in the *XX* spin model with multi-site interaction^18^. It is known that the origin of nonanalyticity in the concurrence at Δ_*f*_ comes from the requirement that the concurrence should be non-negative instead from the nonanalyticity of *ρ*. Therefore, the discontinuity in 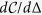 does not necessarily indicate the existence of QPT. In Fig. 5(b) and (c), dependences of Hellinger distance 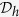 and quantum discord 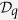 on Δ at several temperatures with *h* = 0 are plotted. Obviously, both of them exhibit a cuspate behavior when the anisotropy parameter Δ increases. These phenomena cannot appear under the influence of magnetic field. For the QD, such phenomena can be also observed in other models^46^. Thus we see that the first derivative of these quantum correlations would show discontinuities at both points Δ_*c*_ = 1 and Δ_*p*_. The discontinuity at Δ_*c*_ = 1 does indicate the QPT of the present model. However, the unexpected discontinuity point occurring at Δ_*p*_ is not a real critical one. In comparison with the behaviors of above typical quantum correlations, we also plot the trance distance discord in Fig. 5(d). Obviously, the 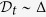 curve is continuous and smooth, and we can anticipate that there would be one point where the first derivative of 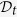 is discontinuous as temperature approaches to zero, marking the critical point exactly. Therefore, we can reasonably state that the trace distance, in contrast to other quantum correlations (e.g., concurrence, quantum discord and Hellinger distance), may be more reliable to spotlight the critical points for this model under certain situations at finite temperature.

### Quantum criticality from ENQ to UFM

**Figure 6:**
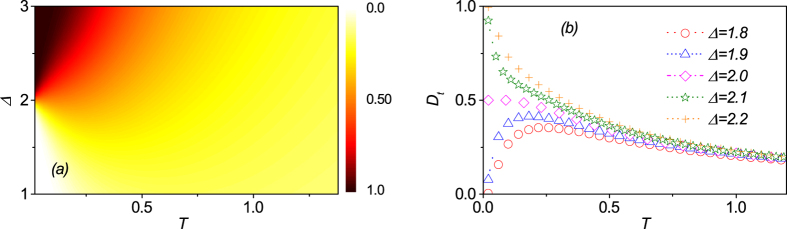
(**a**) The trance distance discord 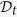 as a function of *T* and Δ. (**b**) The trance distance discord 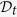 as a function of *T* at several different values of parameter Δ.

Based on the above discussion, one understands that there exists another transition from ENQ phase to UFM phase around critical line Δ − 2*h* + 3 = 0 with *h* > 2. Figure 6(a) and (b) present the 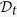 as a function of parameters Δ and *T* for a fixed external field *h* = 2.5. The 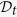 pattern is very similar to the counterpart around critical line Δ = 1 with *h* < 2. For instance, 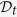 decreases monotonically with increasing *T* and approaches to zero at high temperature when Δ > 2.0. For Δ < 2.0, 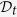 is nearly zero in the low temperature limit, but increases rapidly to a maximal and then falls gradually down to zero again with increasing *T*. These results also suggest that the system undergoes a QPT as parameters Δ or *h* pass across the critical line Δ − 2*h* + 3 = 0 with *h* > 2 at finite temperature.

**Figure 7:**
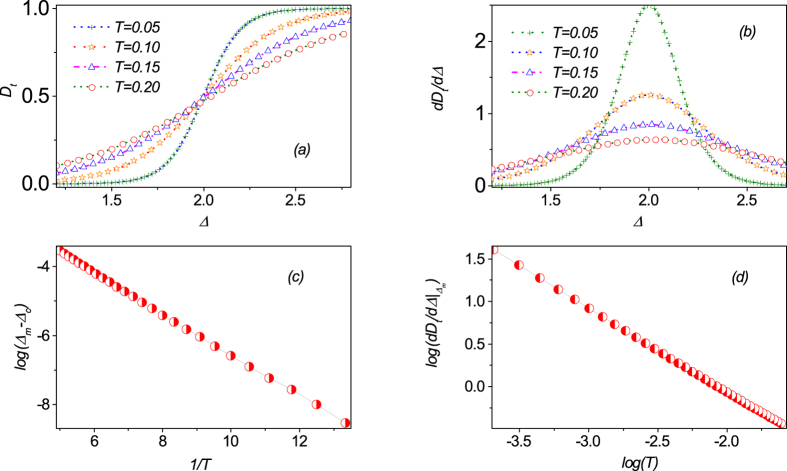
(**a**) 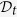 and (**b**) its first-derivative 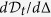 as a function of parameters Δ, respectively, around the critical line Δ − 2*h* + 3 = 0. (**c**) The peak position Δ_*m*_ can be regarded as a pseudo-critical point which shifts with temperature *T* following relation 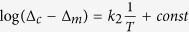 in approaching to the critical point Δ_*c*_. This character implies Δ_*m*_ → Δ_*c*_ as *T* → 0. (**d**) The maximal value of 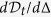 at the pseudocritical point Δ_*m*_ as a function of *T*. The scaling behavior is very different from the counterpart in the quantum criticality from ENQ to UFI.

To further understand the properties of 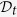 around the critical line Δ − 2*h* + 3 = 0, we investigate the finite-temperature scaling behavior quantitatively. In Fig. 7(a), we present 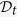 with respect to Δ around the point (Δ, *h*) = (2.0, 2.5) at different temperatures *T*. 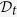 increases monotonically with increasing Δ and this dependence becomes more significant at lower temperature *T*. To characterize this dependence, we also plot the first-derivative of 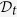 with respect to Δ in Fig. 7(b), and a singularity around the critical point (Δ, *h*) = (2.0, 2.5) in the limit *T* → 0 is displayed. One also observes that the peak position marking the anomaly shifts and the peak height decreases with increasing temperature, and the peak position Δ_*m*_ as a pseudo-critical point can be described by the following scaling law:


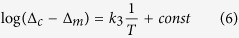


in approaching to the critical point Δ_*c*_. The numerical results are plotted in Fig. 7(c), implying Δ_*m*_ → Δ_*c*_ as *T* → 0. On the other hand, at the pseudo-critical point Δ_*m*_, the value 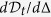 diverges logarithmically with decreasing temperature *T*, according to


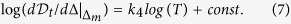


The numerical results are plotted in Fig. 7(d). Here, it should be mentioned that the scaling behaviors are very different from the counterpart in the quantum criticality from ENQ phase to UFI phase. The two kinds of transitions can be distinguished by the different finite-temperature scaling behaviors.

**Figure 8:**
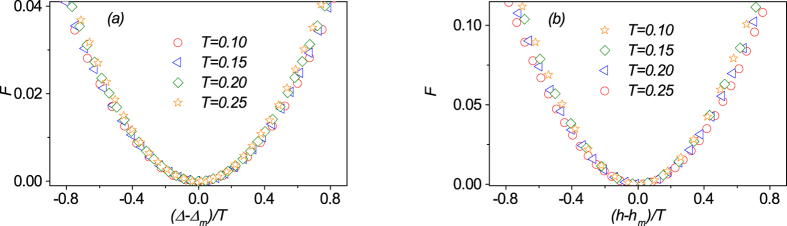
(**a**) The evaluated 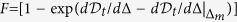 as a function of (Δ − Δ_*m*_)/*T* at different temperatures. (**b**) The evaluated 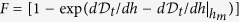 as a function of (*h* − *h*_*m*_)/*T* at different temperatures. Given the fixed parameter *h* = 2.5 (**a**) or Δ = 2 (**b**), all the data collapse on a single curve, respectively, as expected from the finite temperature scaling ansatz.

Furthermore, by proper scaling and taking into account the distance of the maximum of 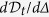 from the critical point, all the data at different temperatures can be properly re-scaled onto the single curve using the scaling transform relation: 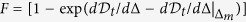 against 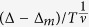
7. The results around the point(Δ, *h*) = (2, 2.5) are plotted in Fig. 8(a), demonstrating the scaling of the critical behaviors. The critical exponent *ν* = 1 is obtained.

We understand that the transitions across the critical line Δ − 2*h* + 3 = 0 can be driven by Δ or *h*. It is also well known that the most important ingredient of physics with quantum phase transitions is the universality class, which means that different driving parameters may exhibit the same behavior around the critical point and thus have the same critical exponent^1^. To check this universality behavior, we investigate the scaling behaviors given different values of driving parameter, i.e., external field *h*. In our calculations, we find that they do exhibit similar behaviors. We ignore the details and only focus on the results from which the critical exponent can be extracted. By proper scaling treatment and taking into account the distance of the maximal point of 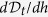 from the critical point, we plot the scaling function 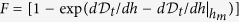 against 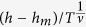 for different temperatures *T* around the point(Δ, *h*) = (2, 2.5) in Fig. 8(b). Obviously, all the data collapse onto a single curve, confirming the scaling behavior. The extracted critical exponent is *ν* = 1. These results also demonstrate convincingly that the quantum critical behaviors can be characterized by the trace distance even at nonzero (finite) temperature.

## Discussion

Here the quantum criticality in the Ising-XXZ diamond structure at finite temperature have been studied by the trance distance discord calculations. Around the critical lines, the first-order derivative of the trace distance discord exhibits a maximal at a finite temperature and diverges under the thermodynamic limits *T* → 0. By analyzing the finite-temperature scaling behaviors, we show that the trace distance discord can detect exactly the quantum phase transition from the entangled state in ferrimagnetic phase to an unentangled state in ferrimagnetic phase or to an unentangled state in ferromagnetic phase. The results also show that the trace distance can distinguish the two kinds of transitions by consulting to the different finite-temperature scaling behaviors. As a comparison, we also study the behaviors of some other typical quantum correlations (e.g., concurrence, quantum discord and Hellinger distance) around the critical points, and the results state that the trance distance discord is more reliable than the others to spotlight the critical points for this Ising-XXZ diamond structure at finite temperatures.

Surely, this model system has three different critical phases, and it would be significant and challenging in the future to consider the multipartite quantum correlations which may grasp all these transitions. The bipartite quantum correlations imposed on this Ising-XXZ diamond structure, as studied in this work, can not detect the transition from UFI phase to UFM phase at finite temperature, an issue for future investigations.

## Methods

By employing the transfer-matrix method, the reduced density operator for the Heisenberg spin pairs can be obtained exactly^41^,


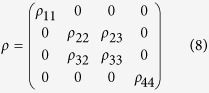


with


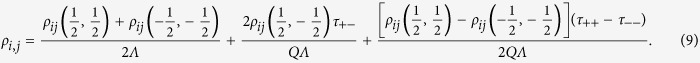


Here, Λ = (*τ*_++_ + *τ*_−−_ + *Q*)/2, 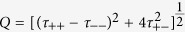, 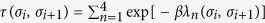 with 
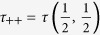
, 
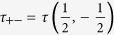
, 
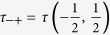
, 
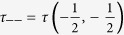
 and *β* = 1/*k*_*B*_*T .T* is the absolute temperature and *k*_*B*_ can be taken as a unit. *ρ*_*ij*_(*σ*_*r*_,*σ*_*r*+1_) are given by,


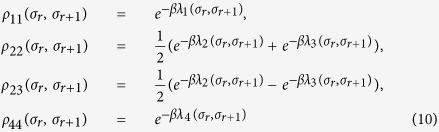


For a bipartite system described by the density operator *ρ*_*AB*_, the trance distance discord is defined as^28^,^29^


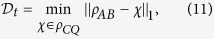


where 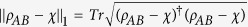 denotes the trace distance between *ρ*_*AB*_ and *χ*∈*ρ*_*CQ*_, and


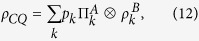


{*p*_*k*_} is the probability distribution, and 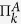 and 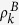 are the orthogonal projector for A and the density operator for B, respectively. For a two-qubit *X* state *ρ*, which only contains nonzero elements along the main diagonal and anti-diagonal, the calculation of the trace distance discord can be simplified by^29^


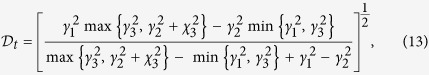


here, *γ*_1,2_ = 2(|*ρ*_23_|±|*ρ*_14_|), *γ*_3_ = 1 − 2(*ρ*_22_ + *ρ*_33_), and *χ*_3_ = 2(*ρ*_11_ + *ρ*_22_) − 1.

## Additional Information

**How to cite this article**: Cheng, W. W. *et al*. Finite-temperature scaling of trace distance discord near criticality in spin diamond structure. *Sci. Rep.* **7**, 42360; doi: 10.1038/srep42360 (2017).

**Publisher's note:** Springer Nature remains neutral with regard to jurisdictional claims in published maps and institutional affiliations.
